# Transcatheter aortic valve implantation for a patient with both severe aortic stenosis and membranous ventricular septal aneurysm: a case report

**DOI:** 10.1093/ehjcr/ytaf230

**Published:** 2025-05-08

**Authors:** Naoto Murakami, Nobuaki Kokubu, Shunsaku Otomo, Masato Furuhashi

**Affiliations:** Department of Cardiovascular, Renal and Metabolic Medicine, Sapporo Medical University School of Medicine, South-1, West-16, Chuo-ku, Sapporo, Hokkaido 060-8543, Japan; Department of Cardiovascular, Renal and Metabolic Medicine, Sapporo Medical University School of Medicine, South-1, West-16, Chuo-ku, Sapporo, Hokkaido 060-8543, Japan; Department of Cardiovascular, Renal and Metabolic Medicine, Sapporo Medical University School of Medicine, South-1, West-16, Chuo-ku, Sapporo, Hokkaido 060-8543, Japan; Department of Cardiovascular, Renal and Metabolic Medicine, Sapporo Medical University School of Medicine, South-1, West-16, Chuo-ku, Sapporo, Hokkaido 060-8543, Japan

**Keywords:** Severe aortic stenosis, Transcatheter aortic valve implantation, Membranous ventricular septal aneurysm, Case report

## Abstract

**Background:**

There have been few reports on transcatheter aortic valve implantation (TAVI) for patients with severe aortic stenosis (AS) and a membranous ventricular septal aneurysm (MSA).

**Case summary:**

A 77-year-old female complaining of dyspnoea was transferred to our hospital. Transthoracic echocardiography (TTE) showed progressive very severe AS with reduced left ventricular (LV) systolic function. The patient was scheduled for TAVI due to high surgical risk. Preoperative computed tomography showed a MSA located between the right coronary cusp and the non-coronary cusp, therefore a part of the annulus rim was lacking. We draw a virtual annulus line to assess her true annulus size and selected a 29 mm size of Evolut Pro Plus. Since the bottom end of the valve was positioned into the MSA, the valve was begun to expand with a lack of coaxiality and massive paravalvular leak (PVL) occurred. Therefore, we decided to retrieve the 29 mm valve. An up-sized 34 mm Evolut was tried, but it was too large and caused the phenomenon of stent-frame infolding. We had to retrieve the 34 mm valve again, and tried to deploy another 29 mm valve at a position as high as possible and pushed the delivery system during the final release to maintain good coaxiality. Postoperative TTE showed significant recovery of LV systolic function, and the PVL was mild.

**Discussion:**

In patients with both MSA and severe AS, it is difficult to measure the precise annulus size for ensuring stability of the self-expanding valve and preventing PVL.

**Learning points**

- In patients with aortic stenosis and a membranous ventricular septal aneurysm (MSA), it might be difficult to estimate precise annulus area excluding the MSA and select an appropriate valve size.
- A self-expanding valve that can cover a wide range of annulus sizes and has the advantage of being repositionable and retrievable is suitable for such patients.

## Introduction

Transcatheter aortic valve implantation (TAVI) has been established as an effective and safe treatment for patients with severe aortic stenosis (AS). With the expansion of TAVI indication, patients with various characteristics might have a treatment opportunity. Nevertheless, there have been relatively few reports on TAVI for patients with congenital heart diseases.^1–4^ A membranous ventricular septal aneurysm (MSA) is a rare condition, and its aetiology is not completely understood. Here, we present a case report of TAVI for a patient with severe AS coexisting with MSA.

## Summary figure

**Table:** 

Time	Event(s)
Admission	Transferred from previous hospital
Day 2	Echocardiography revealed very severe AS and LV dysfunction.
Day 7	CT scan revealed membranous septal aneurysm between the RCC and NCC.
Day 16	TAVI using SEV performed in the patient
Day 17	Dobutamine was discontinued.
Day 21	Echocardiography showed haemodynamic improvement.
Day 30	Transferred to the previous hospital

## Case presentation

**Figure 1:**
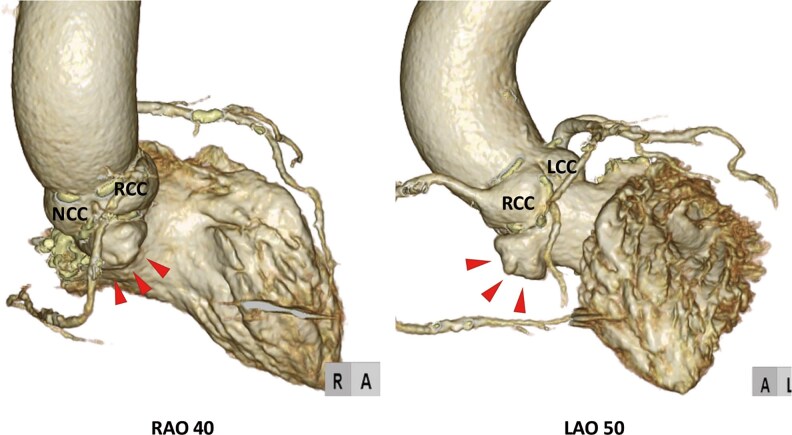
3D images of membranous septal aneurysm.

**Figure 2:**
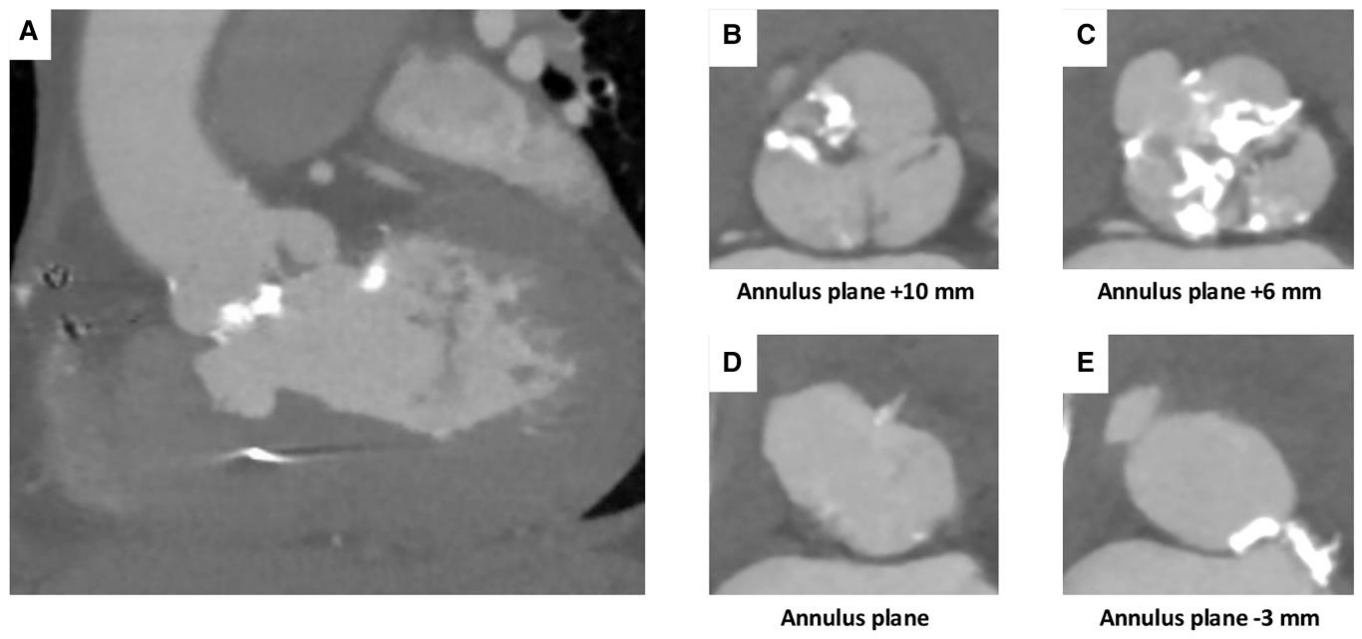
Cross-sectional views of membranous septal aneurysm. (*A*) Vertical view of the membranous septal aneurysm. (*B*) Annulus plane +10 mm. (*C*) Annulus plane +6 mm. (*D*) Annulus plane. (*E*) Annulus plane −3 mm.

**Figure 3:**
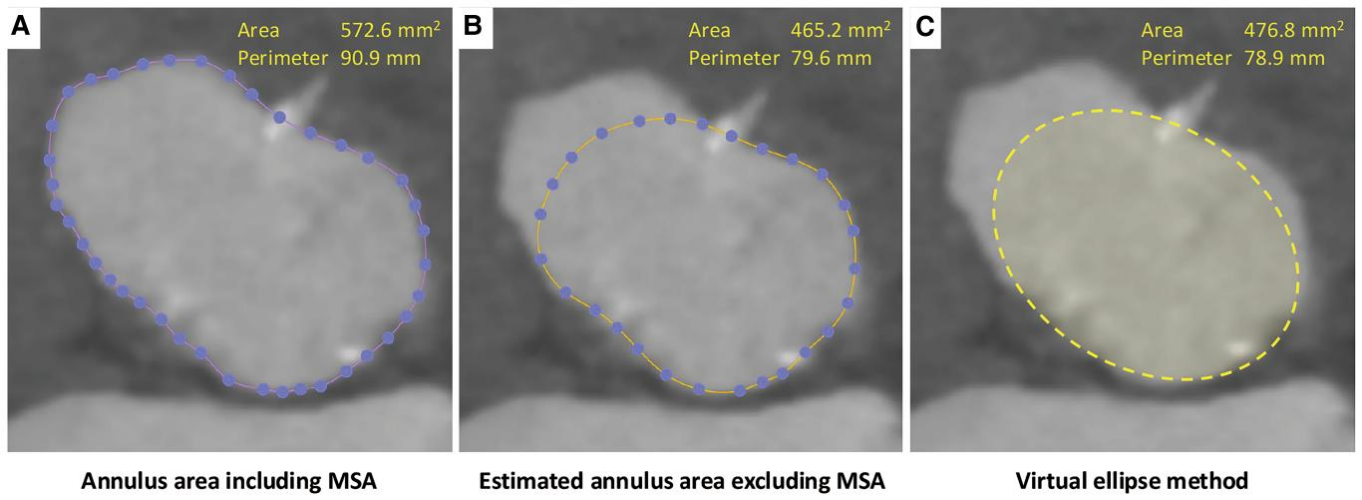
Annulus measurements with and without membranous septal aneurysm. (*A*) The largest estimated value of the annulus including membranous septal aneurysm. (*B*) Feasibly estimated value of the annulus excluding membranous septal aneurysm. (*C*) Evaluation based on the virtual ellipse method.

**Figure 4:**
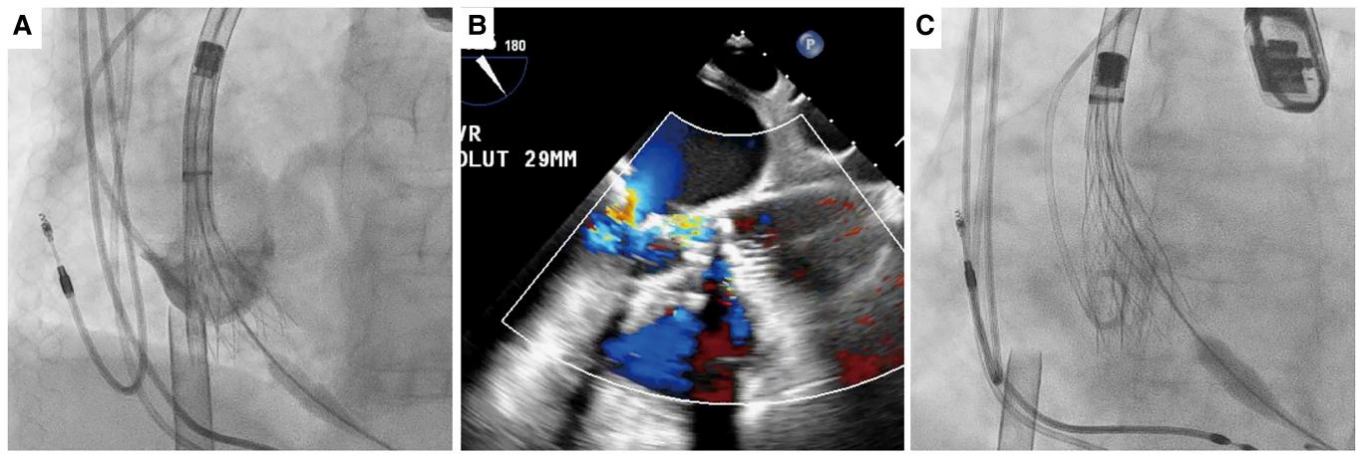
(*A*) The first 29 mm valve resulted in massive paravalvular leakage due to a lack of coaxiality. (*B*) Transoesophageal echocardiography image of paravalvular leakage with the first 29 mm valve. (*C*) The next 34 mm valve caused stent-frame infolding.

**Figure 5:**
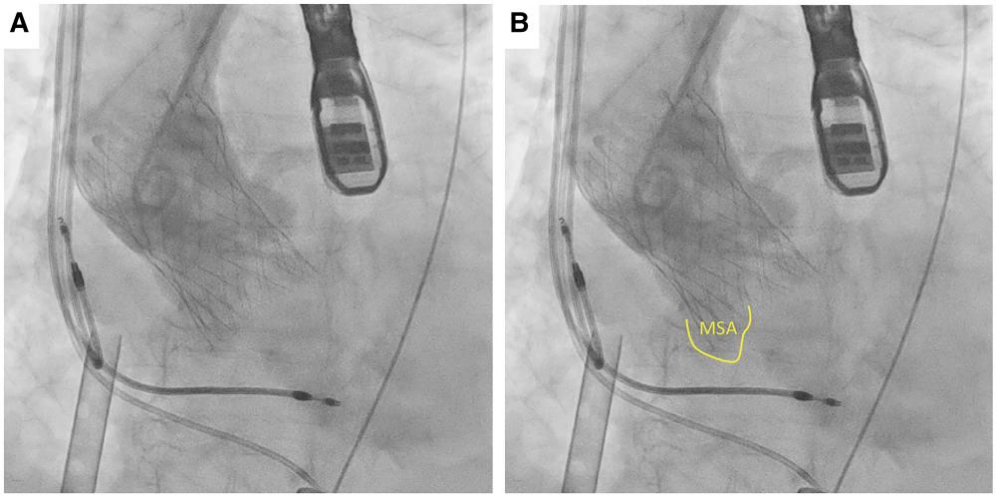
(*A*) Final angiogram post-29 mm valve deployment. (*B*) Illustration of the membranous septal aneurysm location.

A 77-year-old female complaining of dyspnoea on effort was admitted to the previous hospital. Transthoracic echocardiography (TTE) revealed very severe AS with left ventricular (LV) systolic dysfunction, of which peak velocity was 5.0 m/s, peak and mean aortic valve gradient were 97.7 and 61.6 mmHg. Her LV wall motion was generally hypokinetic, and the left ventricular ejection fraction (LVEF) was 31%. She had concomitant moderate mitral stenosis, but no other significant valvular disease was observed. And her estimated systolic pulmonary artery pressure was 29 mmHg. She presented congestive heart failure; however, pulmonary congestion disappeared with continuous dobutamine infusion and tolvaptan 7.5 mg/day. The patient was transferred to our hospital to receive AS treatment. She had a systolic ejection murmur, but no rales were heard. Her NT-pro BNP level was elevated at 21 231 pg/mL, serum creatinine was 1.56 mg/dL, and haemoglobin was 11.4 g/dL. The Society of Thoracic Surgeons predicted that risk of mortality was 7.30% and our heart team decided to perform TAVI instead of surgical aortic valve replacement. A preprocedural computed tomography (CT) scan revealed MSA between the right coronary cusp (RCC) and non-coronary cusp (NCC) without any shunt flow (*Figure 1*, Supplementary material online, *Video S1A* and *B*). The continuity of the aneurysmal wall was maintained on CT imaging, and it was considered to be a true aneurysm. The aneurysm extended from 9 mm above to 5 mm below the level of the aortic annulus (*Figure 2*). Because the lack of an annulus rim, it was difficult to measure the precise annulus size. Therefore, we draw a virtual annulus line excluding the aneurysmal area to select valve type and size (*Figure 3B*). We estimated her annulus area and perimeter to be ∼460 mm^2^ and 80 mm, respectively. Due to the uncertainty of true annular measurements and severe calcification on her aortic valve, we decided to use a self-expanding valve (SEV) because of its ability to cover a wide range of annulus sizes and its advantage of being repositionable and retrievable. We selected a 29 mm size of Evolut Pro Plus (Medtronic, MN, USA) for the patient and used extracorporeal membrane oxygenation (ECMO) for haemodynamic stability prior to valve implantation. After the balloon aortic valvuloplasty (BAV) using a semi-compliant balloon of 20 mm in size, we tried to deploy the valve at 3 mm below the NCC, but the bottom of the valve was positioned into the MSA and began to expand with a lack of coaxiality. That resulted in the left coronary cusp (LCC) being uncovered and massive paravalvular leak (PVL) occurred (*Figure 4A* and *B*, Supplementary material online, *Video S2A* and *B*). Therefore, we decided to retrieve the 29 mm valve and upsize to a 34 mm valve. We determined that the appropriate annular measurement for the SEV should be considered as the annular area including the MSA, of which the perimeter was 90.9 mm (*Figure 3A*). We therefore decided to use a valve of 34 mm in size, but it was too large and caused the phenomenon of stent-frame infolding (*Figure 4C*, Supplementary material online, *Video S2C*). Since transoesophageal echocardiography also showed valve underexpansion, we had to retrieve the 34 mm valve once again. Before trying a brand-new 29 mm valve, BAV using a non-compliant (NC) balloon of 22 mm in size was performed. Then, we started to deploy another 29 mm valve at a position as high as possible and controlled the delivery system direction during the final release to tilt the valve. After post-dilatation with the 22 mm NC balloon, the PVL was trivial (*Figure 5*, Supplementary material online, *Video S3*). The ECMO was successfully retrieved immediately after the TAVI procedure. Postoperative TTE on the fifth postoperative day (POD) showed significant LVEF recovery from 31% to 51% and mild PVL around the LCC. The postoperative peak and mean pressure gradients were low, measuring 21.9 and 6.9 mmHg, respectively. Dobutamine infusion was discontinued on POD 1, and no other vasoactive agents were administered. The diuretics were discontinued after the TAVI procedure, and bisoprolol 1.25 mg/day was initiated. The patient was transferred to the previous hospital on POD 14, and was admitted to a care facility approximately one month later.

## Discussion

Membranous ventricular septal aneurysm is an aneurysmal formation occurring in the membranous septum, which is located below the annulus between the NCC and RCC, and it is often incidentally detected by TTE or CT imaging. It is a rare congenital disease and is associated with spontaneous closure of a membranous ventricular septal defect. It has been reported that the incidence of MSA is ∼0.3% in patients with all congenital heart diseases.^5^ Although the aetiology of MSA is poorly understood, weakness of the membranous septum lacking muscle tissue might cause the development of MSA.^6^ Previous report also indicated that the membranous septum morphology may associated with conduction abnormalities after TAVI, and in particular, the difference of the membranous septum edge and the lower edge of the transcatheter heart valve has been reported to predict the incidence of conduction abnormalities.^7^ In this case, the aneurysm existed around the RCC–NCC commissure, extending across both the aortic and LV sides of the annulus plane. Since the structure of the Valsalva sinus was preserved as normal, it was determined to be a septal aneurysm that extends partially to the aortic side, rather than a Valsalva sinus or annular aneurysm. The nadirs of the three native aortic cusps were present as usual, allowing us to identify the annular plane and central axis of aortic complex. However, because of the lack of annular continuity, annulus measurement might be a crucial problem in planning TAVI. For the selection of valve size, a virtual simulation by drawing an ellipse at the annulus plane (*Figure 4C*) may be useful to determine the prosthetic size, similar to the ‘circle method’ in a case with bicuspid aortic valve.^8^ Although BEVs were commonly chosen in previous case reports, an SEV was selected in this case because it was considered to be able to cover a wide range of annulus sizes. However, floating of the lower edge below a cusp causes difficulty in maintenance of the device coaxiality and does not provide optimal coverage at the aortic annulus and can lead to massive PVL. Moreover, the patient is relatively young at 77 years old, and selecting SEV raises concerns about losing the future repeatability of TAVI. If the valve size were not borderline between 29 and 34 mm, a balloon expandable valve could also be considered.

In summary, in patients with both MSA and severe AS, it is difficult to measure the precise annulus size for ensuring stability of the SEV and preventing PVL. This case indicates the importance of careful planning and deployment with fine adjustment of the SEV in patients with above anomaly.

## Patient’s perspective

The patient and her family were concerned about the high procedural risk of surgical aortic valve replacement and actively chose TAVI as the treatment option.

## Lead author biography


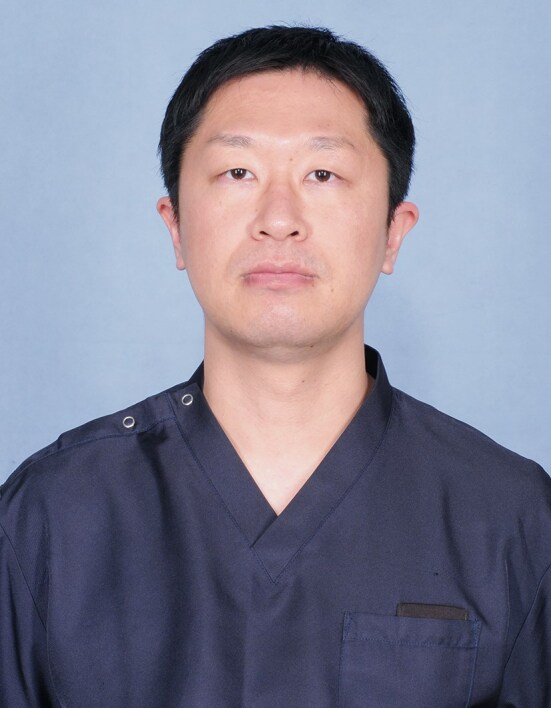


Naoto Murakami is an interventional cardiologist and assistant professor in the Department of Cardiovascular, Renal and Metabolic Medicine at Sapporo Medical University Hospital. His research focuses on structural heart disease, coronary artery disease, and out-of-hospital cardiac arrest.

## Supplementary Material

ytaf230_Supplementary_Data

## Data Availability

The data underlying this article are available in the article and in its online Supplementary material.

## Supplementary material

**Consent:** The authors confirm that written consent for submission and publication of this case report, including images and associated text, was obtained from the family of the patient in line with the COPE guidelines.

**Funding:** The authors did not receive any funding for this report.
